# Direct Oral Anticoagulant Use and Risk of Diverticular Hemorrhage: A Systematic Review of the Literature

**DOI:** 10.1155/2019/9851307

**Published:** 2019-06-18

**Authors:** MacKenzie Turpin, Peter Gregory

**Affiliations:** Department of Medicine, Memorial University of Newfoundland, Medical Education Centre, 300 Prince Philip Drive, St. John's, NL, Canada A1B 3V6

## Abstract

**Background:**

Anticoagulants carry a significant risk of gastrointestinal bleeding. With the increase in use and availability of direct oral anticoagulants (“DOACs”) more data are available regarding the risks of these medications. With diverticular bleeds being common, and hospitalization associated with gastrointestinal bleed increasing 30-day mortality, it is paramount to better understand the potential risks of using DOACs in this population.

**Methods:**

A systematic review of the literature was undertaken, using the databases PubMed, EMBASE, Cochrane Library, and CINAHL. Two reviewers independently searched the literature, and initial screening was performed through title and abstract reading. Search terms included “direct” AND “anticoagulant” AND “diverticular bleed” OR “diverticular hemorrhage”. The references of the selected studies were manually reviewed for any further relevant articles.

**Results:**

Literature search across the databases garnered 182 articles—157 unique abstracts after duplicate removal. Based on inclusion and exclusion criteria, 6 studies were deemed relevant. The selected studies' reference lists yielded no further relevant articles.

**Discussion:**

Across the 6 studies, the incidence of diverticular bleeding in patients using DOACs was extremely low. Of 23,990 patients taking DOACs identified from two separate institutions, only 60 were found to have diverticular hemorrhage. Similarly, among 15,056 patients with diverticular hemorrhage, only 246 (1.6%) among them were taking DOACs. Generally, the studies found no increased diverticular bleeding rate between patients taking DOACs and those who were taking other anticoagulants, such as warfarin, or the general population. The studies also did not find an increased risk of rebleeding with DOAC continuation.

**Conclusion:**

The evidence suggests the risk of diverticular bleed among DOAC users is equivocal to those not taking DOACs, and the overall incidence of diverticular bleed in the DOAC population is low. As it stands, the risk of thrombotic events from not starting DOACs apparently outweighs the risk of diverticular bleed.

## 1. Introduction

Direct oral anticoagulant medications—also known as DOACs—include among them apixaban, rivaroxaban, and dabigatran. Increasingly, health care professionals and hospitals are turning to these medications as first-line agents in the treatment of venous thromboembolism and atrial fibrillation. Indeed, the Canadian Cardiovascular Society's guidelines for the management of atrial fibrillation suggest DOAC use in preference to warfarin for anticoagulant therapy [1]. However, these agents are certainly not without potential side effects, and studies have shown an increased risk of gastrointestinal bleeding with the use of DOACs [2]. With colonic diverticular bleeding representing the most common cause of lower gastrointestinal hemorrhage, it is pertinent to identify in the population of patients with diverticular disease whether use of DOACs will increase their risk of major bleeding [3, 4]. This is particularly true given the increase in 30-day mortality associated with hospitalization due to diverticular hemorrhage [5]. Thus, this study aims to synthesize the existing literature regarding incidence or risk of diverticular bleed in patients being anticoagulated with DOACs. Ultimately, it will be critical to identify whether these agents are safe for use in patients at increased risk of gastrointestinal hemorrhage.

## 2. Methods

A systematic search for articles of interest was performed in the following databases: PubMed, EMBASE, CINAHL, and the Cochrane Central Register of Controlled Trials. No language restrictions were put in place, and articles were included from inception to August 2018. Example key search terms included “direct oral anticoagulant” or “DOAC” or “novel oral anticoagulant” or “NOAC” or “rivaroxaban” or “apixaban” or “dabigatran” or “edoxaban” AND “diverticular” or “diverticulosis” AND “bleed” or “hemorrhage”.

Two researchers independently and blindly identified and selected the titles, abstracts, and full texts obtained in the database searches. Discrepancies in articles selected were resolved by consensus. The reference lists of included articles were subsequently screened to identify any further articles for inclusion, in accordance with the selection criteria.

Studies were included if they referenced risk of diverticular bleeding (reported as bleeding rate, incidence, odds ratio, or other) in patients taking DOACs for any reason. Studies were excluded if they reported anticoagulant use but were not specific, or gastrointestinal hemorrhage in general without explicit reference to diverticular bleed. Studies were also excluded if they contained the same patient population analyzed through a different lens (i.e., posters presented at a conference as a pilot and then later published as part of a larger study).

## 3. Results

**Figure 1:**
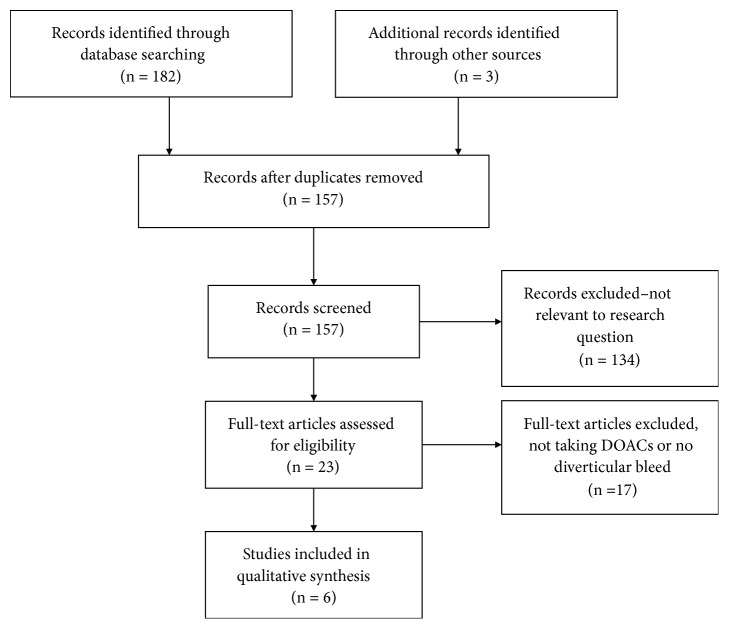
Article selection for inclusion in the systematic review.

The search as outlined identified 182 potential articles for inclusion, and removal of duplicates left 157 unique articles. Through abstract reading, six studies were included in this review that discussed DOAC use in the context of diverticular hemorrhage. The selection process is outlined in Figure 1.

Five of the studies identified were retrospective searches of hospital databases, cross-referencing individuals admitted with a gastrointestinal bleed who were taking DOACs. One article was a prospective study of patients undergoing colonoscopy and identified individuals with evidence of diverticular hemorrhage who were taking DOACs at the time.

The results of the six studies are summarized in Table 1.

This review identified 23,990 patients from two separate institutions taking DOACs of whom only 60 were found to have experienced diverticular hemorrhage, representing a bleeding rate of 0.025%. Similarly, among 15,056 patients discovered to have diverticular hemorrhage, 246 (1.6%) among them were found to be taking DOACs. This is underscored in the statistical analysis performed by Vajravelu et al. which found that anticoagulation with DOACs was not associated with initial or recurrent diverticular bleed [6].

Certainly other risk factors have been well identified in terms of their risk of lower gastrointestinal hemorrhage and may indeed compound the risk of bleeding associated with DOAC use [7]. Unfortunately, the studies by Taki and Kumar did not elucidate whether individual patients were taking multiple agents (such as SSRIs, ASA, or other antiplatelet agents). Nagata's study had insufficient DOAC users to make any conclusions regarding multivariate risk. Vajravelu controlled for potential confounding through the use of Cox modelling and found no significant difference. Brodie's patient population excluded anyone taking clopidogrel or other non-ASA antiplatelet agents and noted that 26 of the DOAC users were taking aspirin. However, only 2 patients taking DOACs had bleeding diverticular and thus no conclusions could be made on any potential associations. Lai's study did determine that DOAC users who experienced gastrointestinal hemorrhage were statistically significantly more likely to be taking clopidogrel SSRIs, yet this study did not demonstrate GI bleeding source. Therefore, based on the studies uncovered in relation to DOAC user and diverticular hemorrhage, it is as-of-yet unclear whether such medications as aspirin and SSRIs put patients at higher risk of bleeding incidence or recurrence.

## 4. Discussion

Use of DOACs has been increasing with the Canadian Cardiovascular Society guidelines favoring their use over warfarin for indications such as atrial fibrillation [1]. Historically, studies investigating bleeding risk through the use of DOACs versus other anticoagulants have been mixed. Select clinical trials such as the EINSTEIN-DVT and -PE studies displayed comparable bleeding risks between warfarin and DOAC (rivaroxaban) use, though the ROCKET-AF trial demonstrated a statistically significant increase in GI bleeding in those patients taking DOACs [12–14]. Of note, none of these trials elicited information on diverticular bleed in particular, though it is the most common source of lower GI bleed. The literature posits that an increased risk of diverticular bleed may stem from an impaired ability for spontaneous bleeding of damaged colonic mucosa to resolve due to the action of the anticoagulant medications, leading to increased patient presentations to hospital for symptomatic bleeding [8].

Studies undertaken to date regarding gastrointestinal bleeding risk—including diverticular hemorrhage—have often overlooked anticoagulants such as DOACs in favor of investigating NSAIDs, antiplatelets, and other agents [3–5, 15–18]. The limited studies that have been undertaken with regard to anticoagulants and gastrointestinal bleeding have typically not specified which drugs were being used. Future studies should clearly highlight the anticoagulant of choice and whether patients are taking any agents that could potentially incite or worsen GI bleeding, such as ASA, other antiplatelet agents, and SSRIs. Furthermore, studies have generally reported bleeding incidence rather than odds or hazard ratios, which limits future meta-analyses. Elucidating such information will be critical as the rates of colonic diverticular bleeding are increasing along with the increase in use of medications such as anticoagulants and antiplatelets [7].

The studies uncovered in this review overall did not indicate increased diverticular bleeding rates in those patients taking DOACs compared to the general population or patients taking other anticoagulant medications. Indeed, Lai et al. found a significantly lower rate of GI bleeding in DOAC users compared to the ROCKET-AF trial (1.7% vs. 3.2%, respectively) and in the 21,503 patient records reviewed found only 52 cases of diverticular bleed across five years of patient records [10]. Further, Taki et al. found similar bleeding rates in both DOAC users and the general population (7% and 3%, respectively) and did not identify a significant difference in bleeding risk between DOAC and warfarin users [8]. Neither the study by Taki et al. nor that by Vajravelu identified a significant association between DOAC continuation and colonic diverticular rebleeding rates [8, 10]. Importantly, Vajravelu et al. found no significant risk of initial diverticular hemorrhage associated with DOAC use but did note that discontinuation of anticoagulation for patients at risk of ischemic stroke resulted in an increased relative hazard ratio for ischemic stroke [6]. With diverticular bleeding being a relatively common phenomenon—and one with high recurrence rates—it is important to bear in mind the potential for adverse sequelae when deciding whether it is appropriate to hold or discontinue such agents as DOACs. In the context of the results of this systematic review, the evidence suggests no increased colonic diverticular bleeding rates amongst DOAC users and thus weighs in favor of continuing these medications for their prophylactic benefits.

## 5. Conclusion

The studies identified herein reported low rates of colonic diverticular bleeding amongst patients taking DOACs. Most commonly, these patients were using DOACs as anticoagulation for atrial fibrillation, in accordance with the most updated version of the CCS Atrial Fibrillation management guidelines. With an increased risk of ischemic stroke when discontinuing DOACs for atrial fibrillation, and no increased risk of recurrent hemorrhage, these studies suggest it is both safe and effective to continue DOACs following resolution of diverticular bleed. Thus, this review lays the foundation to guide clinical decision making in the context of anticoagulation and diverticular hemorrhage. Further studies should investigate timing and dosage of DOAC use in relation to diverticular hemorrhage and proceed with longer follow-up periods to ensure no rebleeding risk. With increased usage of DOACs among the patient population moving forward, such research will be essential to solidifying management guidelines and ensuring patients are effectively and safely anticoagulated.

## 6. Limitations

The studies included did not always distinguish between specific DOAC agent utilized, nor did they identify therapy duration at time of diverticular hemorrhage. The small number of patients taking DOACs in the study by Nagata et al. makes it somewhat difficult to interpret in the context of the remaining studies. Retrospective studies in general have an increased risk of selection bias, and patients may have been omitted from the search results if their primary diagnoses were not accurately input as diverticular hemorrhage (or were simply more generally noted as gastrointestinal bleed) which may have affected the results of the studies herein. Further, low DOAC use rate amongst patients identified in the study—which is most obvious in Nagata et al.—may skew the results. Finally, patients were uncovered only if they presented to hospital with GI bleeding, which therefore overlooks the population of patients with diverticular hemorrhage that did not present to hospital for any reason.

## Figures and Tables

**Table 1:** Summary of reference articles regarding risk of diverticular bleed in DOAC use.

Author and Year	Method	Population Description	Total Population	Population with diverticular bleed	Population with bleed on DOAC (percentage)
Vajravelu et al 2018 [6]	Retrospective cohort study, 2000-2016	Patients with diverticular hemorrhage	14,925	14,925	237 (1.4%)

Nagata et al 2014 [7]	Prospective study 2009-2013	Patients undergoing colonoscopy	911	153	0^*∗*^

Taki et al 2017 [8]	Retrospective case identification 2009-2016	Patients with colonic diverticular bleeding and case-matched controls	300	100	7 (2.3%)

Kumar et al 2015 [9]	Retrospective review all patients in a district hospital	Patients taking DOACS in a district hospital	2,487	8^*∗∗*^	8

Lai et al 2017 [10]	Retrospective review 2010-2015	Patients taking DOACs	21,503	52^*∗∗∗*^	52

Brodie et al 2018 [11]	Retrospective review 2010-2016	Patients with GI bleed	8,496	31	2 (3.3%)

^*∗*^only 2 patients in total were taking DOACs

^*∗∗*^54 total GI bleeds, 14% of which were related to diverticular disease

^*∗∗∗*^366 total GI bleeds
